# A New UPLC-MS/MS Method Validated for Quantification of Jervine in Rat Plasma and the Study of Its Pharmacokinetics in Rats

**DOI:** 10.1155/2019/5163625

**Published:** 2019-03-07

**Authors:** Lianguo Chen, Qinghua Weng, Jianshe Ma

**Affiliations:** ^1^Wenzhou People's Hospital, Wenzhou 325000, China; ^2^School of Basic Medicine, Wenzhou Medical University, Wenzhou 325035, China

## Abstract

The aim of this study was to develop an ultraperformance liquid chromatography-tandem mass spectrometry (UPLC-MS/MS) method to assess the concentration of jervine in rat plasma and its pharmacokinetics. Diazepam was used as internal standard (IS). The chromatographic separation of jervine and IS was carried out on an UPLC BEH C18 column (2.1 mm × 50 mm, 1.7 *μ*m) with a flow rate of 0.4 mL/min. A mixture of acetonitrile and water (0.1% formic acid) was used as a mobile phase. The UPLC-MS/MS was equipped with an electrospray ionization (ESI), adopting multiple reactive monitoring mode to determine jervine in rat plasma. The retention times of jervine and the internal standard were 1.71 and 2.13 min, respectively. The calibration curve of jervine ranged between 1 and 1000 ng/mL. The lower limit of quantitation (LLOQ) was 1 ng/mL, and the lower limit of determination (LLOD) was 0.2 ng/mL. The accuracy was ±6%; the interday precision and intraday precision were no more than 9%. The recovery was higher than 90.3%, and the matrix effect was lower than 10%. The UPLC-MS/MS method was successfully developed and used for the application of the pharmacokinetic study. The primary pharmacokinetic parameters of jervine in this study were as follows: the AUC_(0–∞)_ was 969.3 ± 277.7 ng/mL·h, the *C*_max_ was 506.6 ± 192.8 ng/mL, the CL/F was 1.7 ± 0.5 L/h/kg, and the *t*_1/2_ was 3.4 ± 1.2 h.

## 1. Introduction

Steroidal alkaloids are the principal active and poisonous components in *Veratrum nigrum*, which is a widespread Eurasian species of perennial flower of the family Melanthiaceae in China [1–5]. Jervine, 3-veratroylgermine, veratramine, germanitrine, germidine, and germerine are present in higher content in this plant drug [5, 6]. Recent studies showed that jervine has the potent analgesic properties, antiobese effect, and a significant anti-inflammatory activity against acute inflammation [7–9]. Furthermore, jervine is known as a potent teratogenic agent for the inhibitory effect on the Hedgehog signal pathway [10]. Therefore, in recent years, the antitumor activity of jervine was investigated *in vitro* due to the teratogenic properties [11].

To systematically examine the preclinical pharmacokinetic studies of jervine, a sensitive, fast, and validated analytical method for the determination of jervine in biological fluids is necessary. Previous studies were focus on the extraction, separation, and quantification of jervine and other constituents in Veratrum species using high-performance liquid chromatography (HPLC) [12, 13]. Up to the present moment, only a few bioanalytical methods have been published for the detection of jervine in biological fluids. Lee et al. developed a competitive inhibition enzyme-linked immunosorbent assay (ELISA) for detecting and measuring cyclopamine and jervine using polyclonal antibodies produced in ewes, but the method was complicated, and the results might be prone to false positives [10]. Carlier et al. developed an UPLC-MS/MS method for measuring thirty-four toxic ingredients in the blood, but it is required to validate the method using the required matrix (plasma in this case) [14]. There is only one method for the quantitative and pharmacokinetic analysis of jervine by LC-MS/MS [15], but other five Veratrum steroidal alkaloids (pseudojervine, veratrosine, veratramine, veramarine, and veratroylzygadenine) were also determined simultaneously by means of oral administration of *Veratrum nigrum* extract to rats, and this might influence the pharmacokinetic process of jervine *in vivo*.

Compared with other quantitative methods, the UPLC-MS/MS method has a strong power for the detection and quantification of Chinese traditional herbs [16–19]. Thus, we established an UPLC-MS/MS method to detect the concentrations of jervine in rat plasma and to explore the pharmacokinetic process of jervine for future studies.

## 2. Experimental

### 2.1. Chemicals

**Figure 1:**
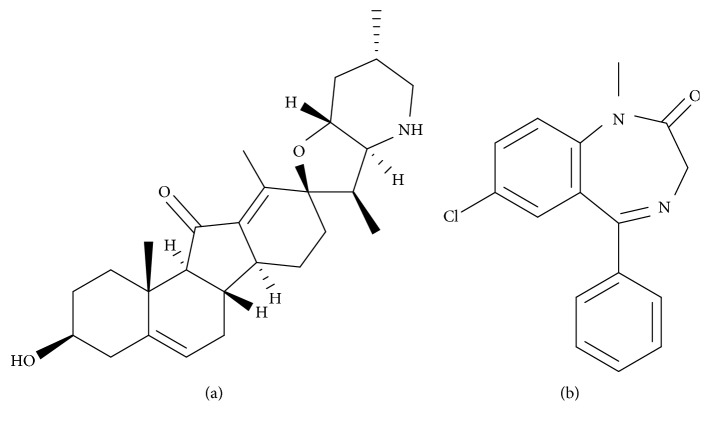
The chemical structure of jervine (a) and diazepam (b).

Jervine (purity >98%, Figure 1) was purchased from Chengdu Munster Biotechnology Co. Ltd (Chengdu, China). Diazepam (IS) was purchased from the National Institute for the Control of Pharmaceutical and Biological Products (Beijing, China). HPLC grade acetonitrile, methanol, and formic acid were provided by Merck Company (Darmstadt, Germany). Ultrapure water was produced by a Milli-Q Water System (Bedford, MA, USA).

### 2.2. Chromatographic and Mass Spectrometric Conditions

An ACQUITY I-Class UPLC system equipped with a XEVO TQS-micro triple quadrupole mass spectrometry (Waters Corp., Milford, MA, USA) was used for separation and detection. MassLynx 4.1 software (Waters Corp.) was used to acquire data and control system.

The separation of analytes was carried out on a Waters UPLC BEH C18 column (2.1 mm × 50 mm, 1.7 *μ*m) at a temperature of 30°C. The mixture of acetonitrile and water (with 0.1% formic acid) was used as mobile phase. The protocol for the gradient eluting with a flow rate of 0.4 mL/min was listed as follows: the concentration of acetonitrile was kept at 10% within 0.2 min, reached 75% within 1.3 min, then kept at 80% from 1.5 to 2.0 min, subsequently decreased back to 10% (2.0–2.5 min), and finally kept at 10% for 1.5 min.

A quantitative analysis of mass spectrometer equipped with electrospray ionization (ESI) source in a positive mode was applied for this determination. The temperatures of the source and drying solutions were set at 150°C and 400°C, respectively. Dry gas (800 L/h) and curtain gas (50 L/h) chambers were filled with high-purity nitrogen. The transitions of *m/z* 426.2 ⟶ 108.9 for jervine (cone voltage was 96 V; collision voltage was 32 V) and *m/z* 285.1 ⟶ 193.3 (cone voltage was 45 V; collision voltage was 36 V) for diazepam were chosen for detection in a multiple reaction monitoring (MRM) mode. The capillary voltage was constantly kept at 2.2 kV.

### 2.3. Preparation of Standards

Standard stock solutions of jervine and diazepam were dissolved in methanol at 1 mg/mL and 0.1 mg/mL, respectively. A batch of working standard solutions was prepared from the stock solution of jervine by diluting with acetonitrile. Internal standard solutions (100 ng/mL) were prepared from the corresponding stock solution of diazepam diluted with acetonitrile. All the solutions were stored at 4°C.

### 2.4. Preparation of Calibration Curves

Calibration standards of jervine were prepared by adding blank rat plasma into appropriated working standard solutions, and the final concentrations ranged from 1 to 1000 ng/mL (1, 5, 10, 50, 100, 200, 500, and 1000 ng/mL). Quality controls (QCs) were prepared in different concentrations (2, 400, and 900 ng/mL) in a similar way. All the solutions were stored at 4°C for subsequent analysis.

### 2.5. Preparation of Plasma Samples

In a 1.5 mL test tube, 50 *μ*L of collected plasma specimen was added to 150 *μ*L acetonitrile (with 50 ng/mL diazepam). After 1 min vortexing, all the tubes were centrifuged at 13000 rpm at a temperature of 4°C for 10 min. Then, a 100 *μ*L of supernatant was transferred into another tube. Finally, 2 *μ*L of supernatant was injected into the UPLC system for the analysis.

### 2.6. Methods Validation

The method validation for this analysis in rat plasma was complied with the guidelines of US Food and Drug Administration (USFDA) [20].

The selectivity was estimated by whether endogenous interference affected the determination of analytes and IS in blank rat plasma. The lower limit of quantification (LLOQ) is the lowest amount of an analyte with a signal-to-noise (S/N) ratio of 10 for the determination of jervine in rat plasma, and final deviation should be within ±20%.

The calibration curve (weighted 1/*x*) was defined as the ratios of peak areas of jervine to that of IS against the levels of jervine in the standard samples at eight different concentrations, including 1, 5, 10, 50, 100, 200, 500, and 1000 ng/mL. The calibration curve was derived from the least squares method for regression analysis. The linearity was estimated by the parameter (correlation coefficient) of calibration curves.

Three different concentrations of QCs (2, 400, and 900 ng/mL) in six replications were prepared for precision in three consecutive days according to the preparation of aforementioned plasma. The interday precision was evaluated by detecting three concentrations of QCs (*n*=6) over different days. The intraday precision was estimated by determining three concentrations of QCs (*n*=6) within the same run. The results were calculated as the relative standard deviations (RSDs) and should be within ±15%. The accuracy was defined as the difference between the theoretical concentrations and the average levels of concentration determined in a similar way. The results were calculated as the relative error (RE) and could not exceed ±15%.

The recovery was calculated as the ratios of the areas of three concentrations (2, 400, and 900 ng/mL) of QCs against those of the standard samples.

The matrix effect was estimated by the ratios of the areas of the plasma samples added three concentrations (2, 400, and 900 ng/mL) to those of the corresponding standard solutions in the mixture of acetonitrile and water with 0.1% formic acid (1 : 1, v/v).

Four conditions were estimated for the stability at three concentrations (2, 400, and 900 ng/mL) of QCs, including plasma added analytes in room temperature for 6 hours, processing plasma samples at room temperature in one day, −70°C for a month, and freeze-thaw cycles for three times (from −20°C to room temperature).

### 2.7. Pharmacokinetic Study

Male Sprague–Dawley (SD) rats (180–220 g) were from Experimental Animal Center of Wenzhou Medical University. The number of the protocol approved was wydw2015-0010. Six rats were given jervine (1.5 mg/kg) by intravenous administration. After administrating, 200 *μ*L of blood samples was separately collected into a 1.5 mL tubes contained heparin from the caudal vein at 0.0833, 0.25, 0.5, 1, 2, 3, 4, 6, and 12 h. All blood samples were centrifuged at 3000 rpm for 10 min and stored at −20°C.

The DAS pharmacokinetic software (version 2.0, China Pharmaceutical University, China) was used to process the main kinetic parameters of jervine [21].

## 3. Results and Discussion

### 3.1. Methods Optimization

Plasma was a kind of complex matrix filled with different endogenous compounds. Therefore, a proper chromatographic method was needed to isolate endogenous substances and analytes [22–24]. In this assay, acetonitrile (0.1% formic acid), acetonitrile and 10 mmol/L ammonium acetate (0.1% formic acid), methanol (0.1% formic acid), and the mixture of methanol and 10 mmol/L ammonium acetate (0.1% formic acid) with a gradient elution were tried. The mixture of acetonitrile and water with 0.1% formic acid was chose in this study for a satisfactory resolution, an acceptable peak shape, and a better retention time.

In order to avoid interference during analysis, most of the endogenous substances and proteins should be removed before the LC-MS/MS analysis [21, 25, 26]. Liquid-liquid extraction (LLE) has the advantages of a high extraction rate and low limit of quantification [27], but the lengthy sample preparation for extraction is intolerable. A one-step protein precipitation procedure was chosen in our study following the example of previous studies, and the methanol, acetonitrile, and the mixture of methanol and acetonitrile (1 : 1, v/v) were tested [21, 28]. Finally, protein precipitation by acetonitrile significantly simplified the sample preparation and showed a better recovery of extraction and an acceptable matrix effect. The LOQ for jervine (1 ng/mL) in our study is low enough for detection. Therefore, direct precipitation by acetonitrile was chose to prepare the plasma samples.

For this analytical method, diazepam was selected as the IS for its stability, chromatographic, and extraction behaviors.

### 3.2. Methods Validation

**Figure 2:**
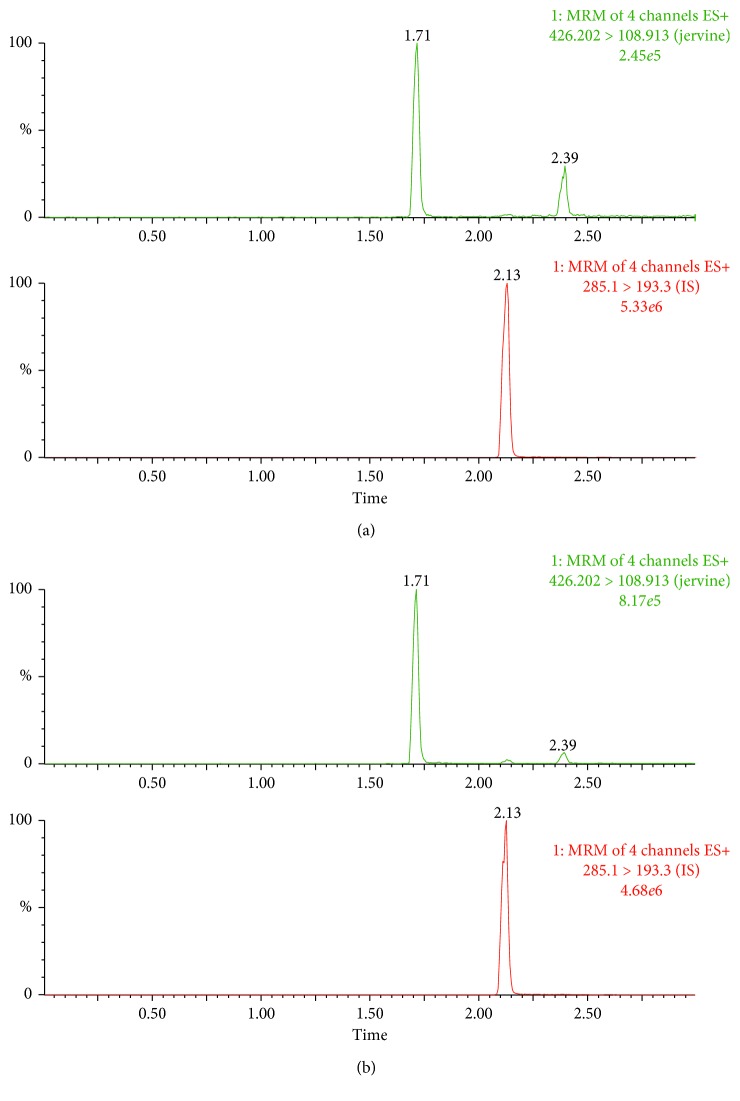
UPLC-MS/MS of jervine and diazepam in rat plasma. (a) The blank plasma spiked with jervine and diazepam and (b) the plasma samples after intravenous administration of jervine.

The spectrogram of blank plasma with the addition of jervine and IS, and casual plasma samples derivated from vein after administration of jervine is shown in Figure 2. The retention times of jervine and IS were 1.71 and 2.13 min, respectively. There was no obvious interference found at the retention time of jervine and IS.

The equation of the calibration curve of jervine in this study was *y* *=* 0.007021*x + *0.000632 (*r*=0.9992), where *x* is the levels of jervine in plasma and *y* is the ratios of area of jervine against IS. The linearity of the calibration curve was great from 1 to 1000 ng/mL. The LLOQ was 1 ng/mL; the corresponding precision and accuracy were 10.2% and 93.8%. The limit of detection was 0.2 ng/mL (signal-to-noise ratio was 3).

The precision, accuracy, recovery, and matrix effect of jervine are presented in Table 1. The accuracy was from 95.2% to 104.8%, the interday precision was less than 9%, and the intraday precision was less than 7%. The recovery was above 90.3%, and the matrix effect was between 91.6% and 104.4%. The variations of stability were within ±10%, and RSD less than 11%, which showed that jervine inhibited great stability in the four different conditions described above.

### 3.3. Pharmacokinetic Study

**Figure 3:**
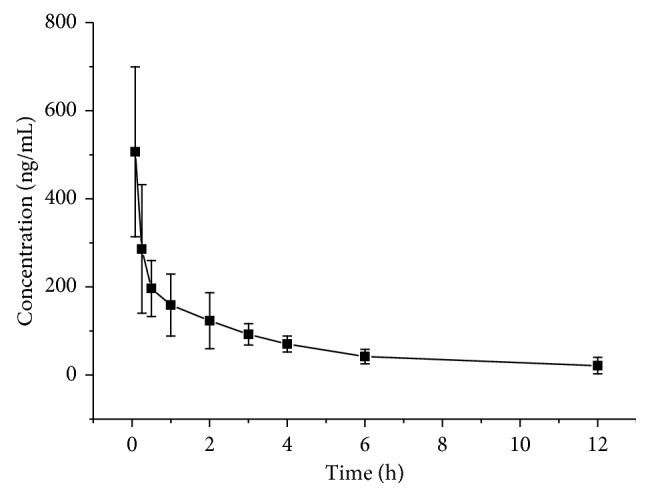
The mean plasma concentration-time profile after intravenous administration of jervine (1.5 mg/kg).

The time-concentration profile of jervine by intravenous administrations is presented in Figure 3. As shown in Table 2, the pharmacokinetic parameters were estimated by the noncompartment model (detailed results listed).

Chen et al. used a sensitive hydrophilic interaction liquid chromatography (HILIC) electrospray ionization mass spectrometry for the simultaneous determination of six steroidal alkaloids and applied it to pharmacokinetic study after oral administration of *Veratrum nigrum* extracts to rats [15]. The primary pharmacokinetic parameter of jervine in their study was as follows: the CL/F was 4.8 ± 0.5 L/h/kg and the *t*_1/2_ was 0.9 ± 1.3 h. Comparing with these results, in our study, the CL/F was decreased by 64.6% and the *t*_1/2_ was extended by 2.8 times, showing that the metabolism of jervine was obviously decreased without those another five steroidal alkaloids isolated from the *Veratrum nigrum* extract, especially the other five steroidal alkaloids. A follow-up research is needed because the increased content of jervine *in vivo* might increase the toxicity.

The drug metabolic interaction generated by the induction or inhibition of Chinese herbal medicines on hepatic drug-metabolizing enzymes (CYP450), UDP-glucuronosyltransferase (UGT), or drug transport protein is very common [29–31]. It has been found that *Veratrum* species contain over than 200 different alkaloids, besides flavonoids and stilbenoids constituents [6, 32]. However, there are no reports on the interaction between jervine and other components. In order to avoid the interference from the complex constituents of isolated extract and explore the real pharmacokinetic characteristics of jervine, we chose to administer jervine directly to rats and determine it in plasma.

## 4. Conclusion

In this present assay, a rapid, sensitive, and selective UPLC-MS/MS method was established to detect the concentration of jervine in rat plasma. This method was finally successfully applied for the pharmacokinetic study of jervine after intravenous administration, and it is suitable for further studies on drug-drug interaction.

## Figures and Tables

**Table 1:** Accuracy, precision, matrix effect, and recovery of jervine in rat plasma.

Concentration (ng/mL)	Accuracy (%)		Precision (RSD %)		Matrix effect (%)	Recovery (%)
	Intraday	Interday	Intraday	Interday		
2	7.4	4.9	95.2	103.5	91.6 ± 7.9	95.0 ± 8.4
400	3.9	5.0	97.6	97.9	97.3 ± 1.8	90.3 ± 1.7
900	8.5	6.4	100.5	104.8	104.4 ± 6.6	97.2 ± 4.8

**Table 2:** The main pharmacokinetic parameters of jervine after intravenous administration.

Parameters	Unit	Jervine
AUC_(0–*t*)_	ng/mL·h	892.6 ± 285.0
AUC_(0–∞)_	ng/mL·h	969.3 ± 277.7
MRT_(0–*t*)_	h	3.2 ± 0.6
MRT_(0–∞)_	h	4.4 ± 1.5
*t*_1/2z_	h	3.4 ± 1.2
CL_z/F_	L/h/kg	1.7 ± 0.5
*V*_z/F_	L/kg	8.1 ± 4.0
*C*_max_	ng/mL	506.6 ± 192.8

## Data Availability

The data used to support the findings of this study are included within the article.
